# Development of microsatellite molecular markers and genetic diversity in *Hevea Braziliensis*

**DOI:** 10.1186/1753-6561-5-S7-P11

**Published:** 2011-09-13

**Authors:** Camila Campos Mantello, Fernando Issamu Suzuki, Livia Moura Souza, Paulo de Souza Gonçalves, Anete Pereira de Souza

**Affiliations:** 1 Molecular Genetic Analysis Laboratory - Molecular Biology Center and Genetic Engineering (CBMEG), UNICAMP, Campinas, São Paulo, 13083-875, Brazil; 2 Agronomy Institute of Campinas (IAC), Campinas, São Paulo, 13012-970, Brazil; 3 Molecular Genetic Analysis Laboratory - Molecular Biology Center and Genetic Engineering (CBMEG), UNICAMP, Campinas, São Paulo, 13083-875, Brazil and Department of Botany, Biology Institute, UNICAMP, Campinas São Paulo, 13083-970, Brazil

## Background

The rubber tree [Hevea Braziliensis (Willd. ex Adr. De Juss.) Muell-Arg.] is native from the Amazon region which has great economic importance for being the largest source of natural rubber in the world. Although Brazil is the center of origin and genetic diversity of this culture, the country is currently responsible for only 1% of the world production. Besides offering optimal weather conditions for crop development, the Amazon rainforest region is also favorable to the development of the fungus known as SALB (South American leaf blight), which causes the disease-of-leaves. Leaves fall sharply reducing the production of latex, limiting the production of rubber in this region. Thus breeding programs are seeking for clones which are resistant to this fungus and with high production in escape regions, which provide stress conditions such as low temperatures, high altitude, wind and other diseases [1]. The rubber tree is perennial and requires about 30 years to obtain an improved variety, starting from the controlled pollination of a clone to the final recommendation. Molecular markers such as microsatellites (Simple Sequence Repeats, SSRs) are an important tool for diversity studies and potentially to assist breeding programs. This study aimed to develop an enriched microsatellite library for *H. Braziliensis*, characterize these developed microsatellite markers and test the transferability of these markers to six other species of the genus *Hevea*.

## Material and methods

For this study we used 36 accessions of *H. Braziliensis* donated by the Agronomy Institute of Campinas and one accession of each of six other species of genus *Hevea* (*H.nitida*, *H.pauciflora* (*2*), *H.camargoana*, *H.guianensis*, *H.rigidifolia* and*H. benthamiana*) provided by Embrapa. Genomic DNA samples were extracted from lyophilized leaf tissues using a modified CTAB method [2]. Trinucleotide and dinucleotide enriched genomic libraries for *H. Braziliensis* were constructed. The DNA samples were digested with AFAI and enriched using (CT)8 and (GT)8 biotinylated microsatellite probes for the dinucleotide library and (ATC)8 and (CCT)8 for the trinucleotide library. The clones obtained were sequenced and the sequences were evaluated with the Microsat program, which removes parts of the vector and the adapters and verifies the presence of restriction site within the sequence. After this step, the sequences were aligned and edited using the program SeqMan (DNAStar Inc.), which also allows analyzing the redundancy of the library. The identification of microsatellites was performed using a research tool SSRs SSRIT – “The Simple Sequence Repeat Identification Tool” avaiable at Gramene [http://www.gramene.org]and primers complementary to sequences flaking the microsatellites were designed by Primer Select Program (DNAStar Inc) and Primer 3. Amplification tests were made from a temperature gradient to know the annealing temperature and the products were evaluated and resolved on 3% agarose gels stained with ethidium bromide and in denaturing 6% polyacrylamide and silver stained [3]. The loci were characterized on the number of alleles per locus, allele frequency and the Polymorph Information Content (PIC). It was also made analysis of ancestry for de accessions of *H. Braziliensis* using the program Structure v 2.3.3 [4].

## Results and discussion

A total of 384 clones from the dinucleotide library were sequenced while 288 from the trinucleotide library resulting in 133 and 62 microsatellites in each library respectively. It was possible to design 55 and 32 primer pairs for the dinucleotide and trinucleotide libraries respectively. Of the 87 microsatellite loci designed 69 were characterized. The maximum number of alleles per locus was 17 and eight loci were monomorphic. The PIC values ranged from 0.83 to 0.06, the observed and expected heterozygosity ranged from 0.86 to 0.06 to 0.90 and 0.06 respectively. A population analysis divided the 36 accessions into two groups. The cross-amplification of these microsatellites loci was tested in six other species of the genus *Hevea*. The percentage of cross-amplification in others species of *Hevea* ranged from 94.2% to 82.6%, with *H. nitida* with the highest percentage of transferability and *H. carmagoana* with the lowest one. This high percentage indicates that the regions flaking the microsatellites are well conserved between species of *Hevea*. These markers will be useful to breeding and conservation studies of *H. Braziliensis* and related species.

## References

[B1] Pushparajah E Problems and potentials for establishing Hevea under difficult environmental conditions The Planter 1983 59 242 251 10.1111/j.1399-3054.1983.tb00765.x

[B2] Doyle JJ Doyle JL A rapid DNA isolation procedure for small quantities of fresh leaf tissue Phytochem Bull 1987 19 11 15

[B3] Creste S Tulmann A Figueira A Detection of Single Sequence Repeat Polymorphism in denaturating Polyacrylamide Sequencing Gels by Silver Staining Plant Molecular Biology Reporter 2001 19 299 306 10.1007/BF02772828

[B4] Pritchard JK Stephens M Donnelly P Inference of population structure using multilocus genotype data Genetics 2000 155 945 959 10835412 10.1093/genetics/155.2.945 PMC1461096

